# Radiographic Imaging as a Quality Index Proxy for *Brachiaria brizantha* Seeds

**DOI:** 10.3390/plants11081014

**Published:** 2022-04-08

**Authors:** Leonardo Vieira Campos, Arthur Almeida Rodrigues, Juliana de Fátima Sales, Douglas Almeida Rodrigues, Sebastião Carvalho Vasconcelos Filho, Cássia Lino Rodrigues, Dheynne Alves Vieira, Stella Tomaz de Castro, Aurélio Rubio Neto

**Affiliations:** 1 Laboratory of Seeds, Campus Rio Verde, Goiano Federal Institute of Education, Science and Technology (IFGoiano), P.O. Box 66, Rio Verde 75901-970, Brazil; leonardo@zoocampos.com.br (L.V.C.); juliana.sales@ifgoiano.edu.br (J.d.F.S.); douglas.almeida@estudante.ifgoiano.edu.br (D.A.R.); cassialino10@hotmail.com (C.L.R.); dheynne.vieira@estudante.ifgoiano.edu.br (D.A.V.); stella.castro.ab@gmail.com (S.T.d.C.); aurelio.rubio@ifgoiano.edu.br (A.R.N.); 2 Laboratory of Plant Anatomy, Campus Rio Verde, Goiano Federal Institute of Education, Science and Technology (IFGoiano), P.O. Box 66, Rio Verde 75901-970, Brazil; sebastiao.vasconcelos@ifgoiano.edu.br

**Keywords:** image analysis, germination, physical seed quality, X-rays, vigor

## Abstract

Efficient methodologies for automated seed quality evaluations are important for the seed industry. Advanced seed technology research requires the use of adequate methods to ensure good seed performance under adverse environmental conditions; thus, providing producers with detailed, quick, and accurate information on structural seed integrity and ensuring vigorous production. To address this problem, this study aimed to determine *Brachiaria brizantha* (Marandu cv., Piatã cv. and Xaraés cv.) seed quality through radiographic imaging analyses associated with vigor tests and anatomical characterizations. *Brachiaria* seed cultivars displaying different physical and physiological attributes were selected and subjected to the 1000-seed weight test, water content determinations, X-ray analyses, germination tests, and anatomical characterizations. The X-ray analyses made it possible to establish a relationship between the X-ray images and other determined variables. Furthermore, the X-ray images can indicate evidence of internal and external damage that could later compromise germination. The Marandu and Piatã cultivars presented the highest germination percentages, germination speed indices, normal seedling development, and cellular structure preservation compared to the Xaraés cultivar. To summarize, X-ray analyses are efficient methods used for the selection of higher physical quality cultivars and can aid in the decision-making processes of companies and seed producers worldwide.

## 1. Introduction

Grasslands occupy around 80% of agricultural lands worldwide and represent a wide range of ecosystems [1]. In 2015, total global pasture areas comprised 2.7 billion ha, with Africa encompassing the largest area, of approximately 889 million ha, followed by China (~506 million ha), while Brazil holds about 196 million ha of cultivated pasture areas [2]. *Brachiaria* spp., an East African species, also known as signal grass, based on the similarity of its flower head structure with a railway signal, is noteworthy in this scenario [3]. These grasses are used in Brazil for biomass production and as animal feed and soil cover in no-till systems, increasing land use efficiency [4] and soil fertility [5].

As cultivation areas increase, so do demands for high quality seeds, which have become essential for the improvement of the global forage production. Therefore, innovative approaches are required to solve never-before-experienced food production and sustainability problems [6]. This has led the seed industry to constantly improve seed lot production and standardization processes, aiming at obtaining and selling seeds with high physiological potential. In this scenario, seeds used for the establishment of production fields are a fundamental part of this problem and their stability and uniformity are strictly related to the yields obtained at the end of the cycles in many agricultural crops [7].

Some features must be assessed to increase seed lot quality. Seeds may undergo physical characteristic alterations, affecting cell anatomy, including enzymatic inactivation, during the harvesting and storage [8]. Changes at the cellular level can be monitored through germination and emergence rates, electrical conductivity tests, and anatomical techniques. These assessments were employed herein as tools to indicate physiological seed quality, which proved promising, and could bring forth new information on the deterioration of stored seeds.

On the other hand, although traditional tests applied independently in seed quality evaluations produce reliable results, *Brachiaria* seed testing methods are mainly based on the classic tetrazolium test to estimate physiological seed lot potential. This method is, in general, destructive, time-consuming, and influenced by analyst subjectivity. Therefore, new technologies, especially non-destructive high performance, and speedy methods, are required for seed lot assessments.

A high percentage of *Brachiaria* seeds displayed different fillings, directly linked to seed quality, as seed morphology plays a significant role in the growth of healthy and uniform seedlings [9]. Thus, alterations in *Brachiaria* seed development and filling may result in abnormal seedlings. Among other optical technologies, automated X-ray imaging analyses comprise a very interesting tool in this regard due to their high precision, allowing for the detection of damaged or malformed seeds.

X-ray imaging analyses may be employed to evaluate internal seed structures, as well as seed coat features, which allow for physiological performance probability predictions, identifying alterations that indicate the production of abnormal organs and their inevitable consequences during field cultivation, enabling pattern recognition, data management, and providing accurate, fast and non-destructive assessments [10,11]. However, the use of radiographic images for the evaluation of agricultural commodities is still at a preliminary stage, and further studies are required to better determine the quality parameters of seed lots [12].

In this context, this study hypothesized that quality parameters in different *Brachiaria* seed cultivars through imaging analyses are correlated to physiological and anatomical tests results. Thus, the aim of this study was to evaluate the physiological quality of *Brachiaria* seeds employing automated X-ray imaging analyses and compare the findings to germination and anatomical characterizations, in order to quickly and effectively select seed lots.

## 2. Results

### 2.1. Water Content and 1000-Seed Weight

Similar water contents were noted among the three studied cultivars, ranging from 9.1 to 9.8%, with the Marandu cultivar presenting the highest values and Xaraés, the lowest. The 1000-seed weight test results indicated a higher density for the Xaraés cultivar when compared to the other investigated cultivars (damage not shown).

### 2.2. Internal Seed Morphology Assessment Employing the X-ray Technique

**Figure 1:**
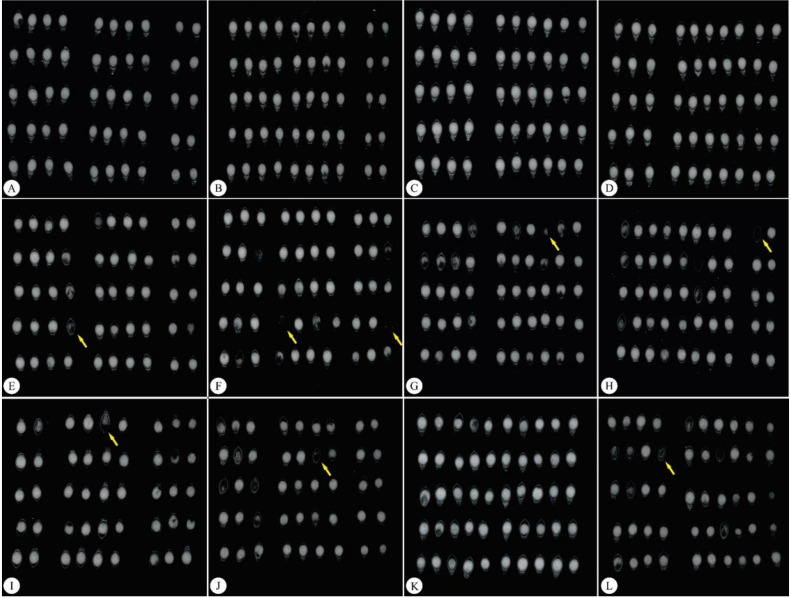
Differences in internal *Brachiaria brizantha* seed structures demonstrated by X-ray assessments. (**A**–**D**) Marandu cv. (**E**–**H**) Piatã cv. (**I**–**L**), Xaraés cv. Yellow arrows indicate damaged seeds.

Seed classifications according to the X-ray technique indicated that the highest number of damaged seeds with endosperm spaces was found for the Xaraés cultivar (Figure 1I–L) compared to the Marandu (Figure 1A–D), and Piatã (Figure 1E–H) cultivars.

**Figure 2:**
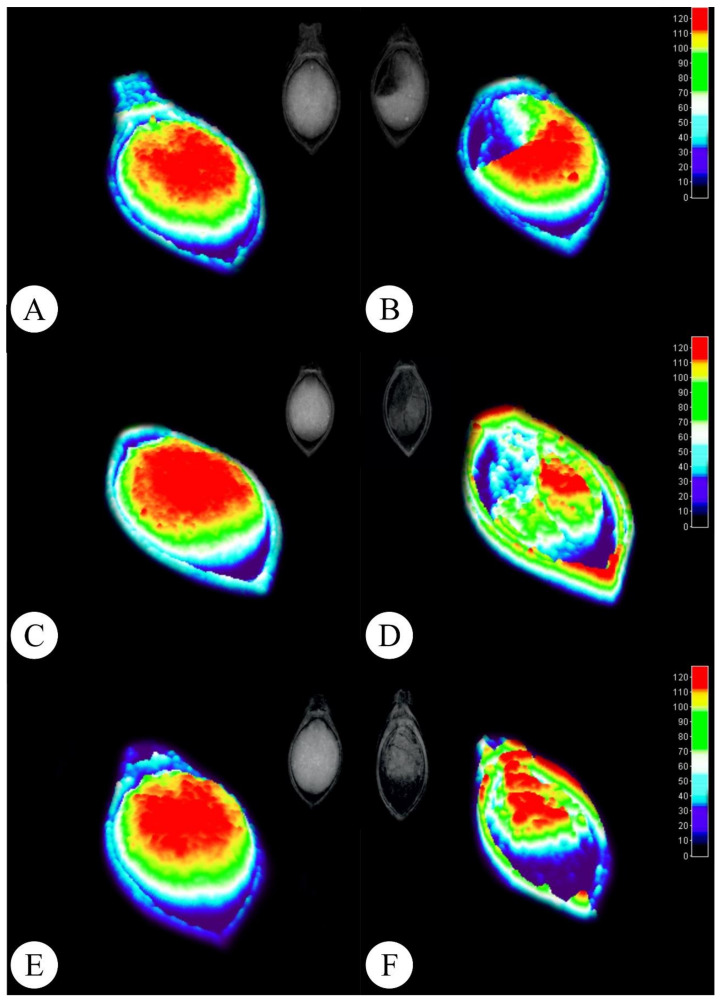
Radiographic *Brachiaria brizantha* seed images and their representations in 3D. (**A**,**B**) Marandu cv. (**C**,**D**) Piatã cv. (**E**,**F**) Xaraés cv. Left column: higher tissue density seeds. Right column: lower tissue density seeds.

The X ray images allow analysts to highlight and select areas presenting physical integrity through grey pixel levels (Figure 2). Figure 2 displays the original X-ray image and its 3D representation with color tone variations according to tissue integrity and density (Figure 2). The 3D histogram technique highlights vigorous regions with warm colors (Figure 2A,C,E), which develop into seedlings, presenting a better development index, while regions with lower physiological quality, which can trigger abnormal seedling development and low germination rates, are indicated in cold colors (Figure 2B,D,F).

Physical seed qualities according to the X-ray images are presented in Table 1. The Marandu cultivar exhibited a greater area and percentage of filling compared to the Piatã and Xaraés cultivars. The Piatã cultivar displayed a similar relative density compared to the Marandu cultivar. The Xaraés cultivar presented the smallest area and lowest relative density and filling percentage compared to the Marandu and Piatã cultivars.

### 2.3. Germination Test

Similar speed index, germination percentage, normal and non-germinated seedling values were observed between the Marandu and Piatã cultivars, with no significant differences (Table 2). However, the Xaraés cultivar exhibited a significant reduction in all analyzed variables compared to the Marandu and Piatã cultivars.

### 2.4. Anatomical Seed Characterization

**Figure 3:**
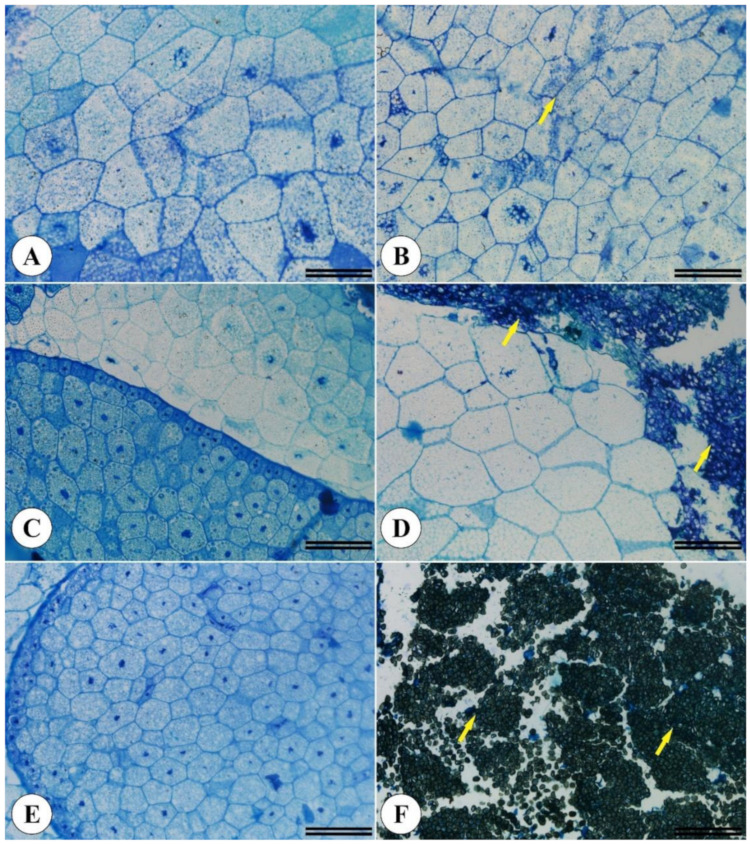
Morphoanatomical *Brachiaria brizantha* seed structures. Seeds previously selected by the X-ray test displaying no filling alterations (**A**,**C**,**E**) and filling alterations (**B**,**D**,**F**). (**A**,**B**) Marandu cv. (**C**,**D**) Piatã vc. (**E**,**F**) Xaraés cv. A total of 200 seeds from each cultivar were analyzed. Yellow arrows indicate cellular changes.

Differences in seed filling led to *Brachiaria* endosperm region alterations (Figure 3B,D,F) compared to seeds displaying no physical changes (Figure 3A,C,E).

**Figure 4:**
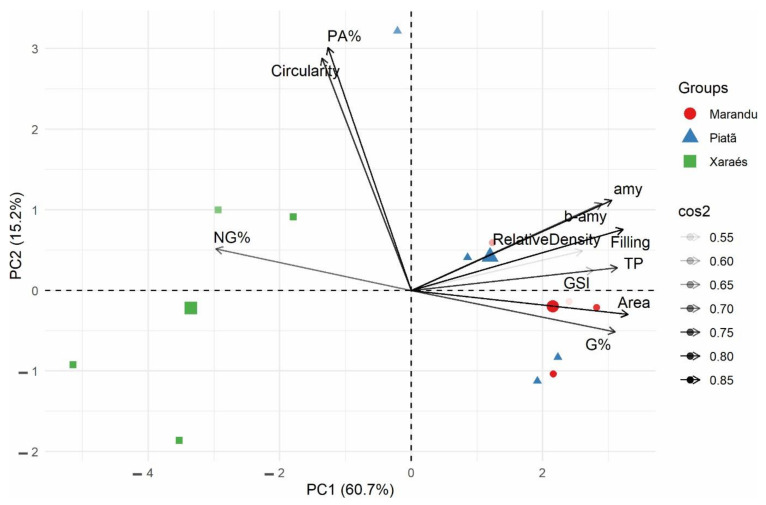
Biplot of the principal components comprising the following variables: area, roundness, density, filling percentage, germination speed index, germination percentage, PT—total proteins, amy = α-amy alpha amylase, b-amy = β-amy beta amylase, normal and abnormal, and non-germinated *Brachiaria brizantha* cultivar seedlings. cos2—importance of the variable in defining the principal component (PC); PC1—first principal component; PC2—second principal component.

The PCA concerning physiological quality variables and quantitative data extracted from the *Brachiaria* seed X-ray images indicate total data variances of 75.40%, 60.20%, and 15.20% for PC1 and PC2, respectively (Figure 4). Three groups of characteristics were detected, namely group I, comprising amy, b-amy, relative density, filling, TP, GSI, area, and G%, positively correlated; group II—PA% and circularity, also positively correlated; and group III—composed of NG%, positively correlated with group II and negatively correlated with group I. Regarding the first component (PC1), seed characteristics NG%, PA%, and circularity exhibited a negative correlation, with NG% as the strongest. The other evaluated characteristics were positively correlated with PC1. Concerning PC2, G% and area were negatively correlated. In general, all characteristics were important for both PC, except for relative density and GSI. Different behaviors are noted regarding the three *Brachiaria brizantha* cultivars (groups), with the Xaraés cultivar antagonistic to the other cultivars and the Piatã and Marandu cultivars exhibiting similar behaviors.

## 3. Discussion

The X-ray image analysis technique can adequately indicate physical seed structure and, as a non-destructive and non-subjective methodology, is highly desired by the seed industry [13]. This methodology allowed for the verification of morphological seed changes caused by several factors, such as physiological changes during the maturation process, pest attacks and mechanical damage during the harvesting and storage processes. The endosperm plays a vital role in supporting embryonic growth, providing nutrients, and protecting and controlling embryo growth, acting as a mechanical barrier during seed development and germination [14]. Therefore, quick, and accurate identification of physical endosperm conditions is important to assess the quality of different seed cultivars.

*Brachiaria* seeds displaying higher density, filling percentages, and cellular integrity exhibited higher physiological quality. These data are paramount for the seed sector, since the quick identification of good and bad quality seeds contributes to optimizing seed production processing and quality control [15,16]. Previous studies on broccoli [17] and leucaena [18] indicate that the determination of seed tissue density employing X-ray images is promising and strongly correlated to physiological seed quality, as higher density seeds are correlated with higher germination percentages and normal seedling development.

Seeds presenting physiological quality changes can undergo important biochemical changes, such as carbohydrate content exudation [19] and oxidation, leading to membrane wall loosening and, as a result, loss of internal protein elasticity, increasing cellular tissue fragility, resulting in cotyledon cracks and irregular development [20]. Furthermore, as evidenced herein, changes in physiological seed quality may be directly linked to seed formation, especially regarding aspects related to cellular disruption in low density seeds with low filling percentages.

Recent research has presented data on internal physical seed parameters evaluated by X-ray images, effectively correlating these data with germination and vigor attributes [17]. In this regard, while studying machine learning to classify *Brachiaria brizantha* seeds, [15] demonstrated that the IJCropSeed tool developed by the researchers is highly efficient in the selection of higher quality seed lots. In the present study, we verified that this relationship may possibly extend to seeds displaying similar physical characteristics when assessing different cultivars of the same species.

The application of the X-ray test on different *Brachiaria* cultivar seeds also indicated an association between internal seed morphology and their physiological quality. This technique thus allows for internal seed morphology characterization, such as identification of full and/or malformed seeds, damage by insect predation, mechanical injuries during harvesting, transportation, and/or storage and percentage of empty internal areas [21,22]).

The use of the X-ray technique is advantageous in reducing seed storage costs by permitting seed separation, contributing to more vigorous seed lots [23]. It also comprises a valuable technique for fast, accurate, and non-destructive assessments related to seed performance. The PCA results corroborate these facts, as seed quality and vigor characteristics were closely associated to parameters determined via the X-ray technique, i.e., area, TP, filling, amy activity, and G%. Furthermore, an opposite NG% behavior compared to these parameters was noted.

Alpha and beta amylase enzymatic activities are directly linked to seed quality, as they can be associated with rapid starch hydrolysis, resulting in greater energy available for initial seedling growth [24,25]. Therefore, amylase analyses also display the potential to assist in the selection of higher quality *Brachiaria* seed lots. In this sense, our findings indicate that seeds presenting higher density, filling, TP, and amy activities form normal seedlings. In addition, differential cultivar behaviors (Xaraés in relation to Piatã and Marandu) also evidence the potential of using the X-ray technique to select seed and cultivar lots.

## 4. Material and Methods

### 4.1. Assay Implementation

All experiments were conducted at the Seed Laboratory, IF Goiano—Rio Verde Campus, GO, Brazil, with uncoated commercial *Brachiaria brizantha* seeds (Marandu cv., Piatã cv. and Xaraés cv.) from the 2020 crop.

### 4.2. The 1000-Seed Weight Test

The weight of 1000 seeds was obtained from eight 1000-seed replicates for each studied *Brachiaria* variety and weighed on a precision scale according to established seed analysis rules [13].

### 4.3. Water Content

Water content was determined by drying the seeds in an oven at 105 ± 3 °C for 24 h, adapted according to [26], using four 50-seed replications, corresponding to 4.5 g for each analyzed *Brachiaria* cultivar. Seeds were weighed on an analytical balance (0.001 g precision) and calculations were performed according to the following equation:

(1) $$Pf=Pi.\left(\frac{100-TAi}{100-TAf}\right)$$

where *Pf*: final sample mass (g); *Pi*: initial sample mass (g); *TAi*: initial seed water content (%wet base); *TAf*: desired water content (% wet base).

### 4.4. Internal Seed Morphology Assessments Employing the X-ray Technique

The X-ray test was performed with 200 Brachiaria seeds, comprising four repetitions of 50 seeds for each cultivar. Briefly, seeds were placed on transparent acrylic plates on double-sided transparent adhesive tape, and subjected to radiation employing a Faxitron HP 43855A X-ray equipment set at 30 Kv for 10 s. The digital images were analyzed using the IJCropSeed plugin developed for the ImageJ^®^ software [7] (Table 3).

After the X-ray seed evaluations, the characterization of the 3D images of the three investigated cultivars was performed using the ImageJ^®^ software. The images were processed in the 3D plugins in the interactive 3D surface plot option, an option that highlights colors in relation to the gray fabric density per pixel. After performing the evaluations, the images were extracted from the software and saved in JPEG format.

### 4.5. Germination Test

The germination test was carried out on sheets of blotting paper moistened with distilled water at 2.5 times the mass of the dry substrate. Four replications comprising 50 seeds each were conditioned in transparent plastic boxes previously washed and dried in a greenhouse. The plastic boxes were maintained in a germination chamber regulated with a photoperiod of 8 h with lighting and 16 h without lighting at a temperature of 35 °C [26].

The percentage of normal germinated seedlings (represented by seedlings displaying all essential structures developed, i.e., root system, shoots, and coleoptile) was computed in the first germination count on the 7th day (G7) and on the final count on the 21st day (G21). The final counts included normal seedlings, abnormal seedlings (those that do not display the potential to continue their development and give rise to normal plants, even growing under favorable conditions), hard seeds (seeds that do not absorb water for a longer period than normal and, thus appear as seeds that have just been placed on the substrate, unswollen at the end of the test), and dead seeds (seeds that are softened, do not exhibit any sign of germination, and do not germinate at the end of the test) [26].

### 4.6. Morphoanatomical Characterizations

Morphoanatomical characterizations were performed by fixing 10 seeds of each *Brachiaria* cultivar according to Karnovsky [27], for 24 h, followed by prewashing in phosphate buffer and dehydration in an increasing ethyl series and, finally, historesin pre-infiltration and infiltration (Leica, Wetzlar, Germany), according to the manufacturer’s recommendations.

For the structural evaluations, samples were cross sectioned at 7 μm thickness on a tabletop rotary microtome (1508R, Logen Scientific, Shanghai, China) and stained with toluidine blue [28]. Observations were performed in the seed reserve region and images were photographed using an Olympus microscope (BX61, Tokyo, Japan) coupled with a DP-72 camera using the brightfield option.

### 4.7. Biochemical Analyses

Enzymatic activities were assessed in 30 whole *Brachiaria* seeds from each cultivar. The seeds were first individually stored in aluminum foil in liquid nitrogen (N_2_) and then in an ultra-freezer at −80 °C prior to further analyses.

The enzyme extracts used to determine α-amylase (α-amy) and β-amylase (β-amy) enzymatic activities were obtained from 0.200 to 0.250 g of frozen seeds homogenized in 2 mL of potassium phosphate buffer (100 mM) (pH 6.8), containing 0.1 mM ethylenediaminetetraacetic acid (EDTA), 5% polyvinylpyrrolidone (PVPP) (m/v), and 1 mM phenylmethylsulfonyl fluoride (PMSF). The homogenates were then kept overnight for 14 h at 10 °C and centrifuged at 12,000× *g* for 15 min at 4 °C. The final supernatants were used as extracts for enzymatic determinations.

Enzymatic activities were determined by the 1% 3,5-dinitrosalicylic acid (DNS) method, as described by Bernfeld [29], Tárrago and Nicolás [30], and Kishorekumar [31]. The reducing sugars formed by the actions of alpha and beta amylase were quantified by determining sample absorbances at 540 nm and calculations were performed using a standard maltose curve at 0.5 mg mL^−1^ 2%.

Total protein concentrations were determined by mixing 10 µL of the crude extracts used to determine the enzymatic activities to 1190 µL of Bradford’s solution and determining sample absorbances at 595 nm Bradford [32], expressed as mg.g^−1^.

### 4.8. Statistical Analyses

The quantitative data were first submitted to homogeneity analysis (Levene’s test) and error normality (Shapiro-Wilk test) evaluations. As data normality was confirmed, an ANOVA test followed by the Scott-Knott test were applied. * indicates *p* < 0.05 and **, *p* < 0.01. Statistical analyses concerning the media test were performed using the software Assistat^®^.

Correlation assessments and a principal component analysis (PCA) were performed employing preliminary database consistency excluding outlier values through a boxplot analysis. The data were then normalized, where the means were equal to 0 and variances, 1. A variance and covariance matrix was then plotted for the PCA analysis [33]. The accumulated total variation was established as a criterion for the selection of the principal components (PC), maintaining the K first PC capable of explaining at least 70% of the total data variation [34].

## 5. Conclusions

X-ray image analyses are associated with physiological and biochemical *Brachiaria* seed quality. Thus, this technique is efficient in the selection of higher quality cultivars and can aid in the decision-making processes of companies and seed producers worldwide. However, despite associations with physiological seed quality, the imaging X-ray technique does not indicate chemical damage or other non-physical damage types, such as genetic effects.

## Figures and Tables

**Table 1:** Physical parameters evaluated from X-ray images of *Brachiaria* seeds, Marandu, Piatã, and Xaraés cultivars, using the ImageJ program.

Cultivars	Area (mm^2^)	Circularity	Relative Density (Grey.Pixel^−1^)	Filling (%)
Marandu	8.33 ± 0.07 a	0.69 ± 0.003 b	70.73 ± 0.69 a	92 ± 0.23 a
Piatã	7.25 ± 0.04 b	0.76 ± 0.003 a	68.31 ± 1.19 a	89 ± 0.58 b
Xaraés	6.63 ± 0.07 c	0.68 ± 0.004 b	58.43 ± 1.18 b	84 ± 0.57 c
**One-Way ANOVA**				
**F (*t*-test)**	193.83 **	132.08 **	38.86 **	76.19 **
***p***	**0.00825**	**0.00000**	**0.00000**	**0.00000**

Means ± SE (*n* = 4), Means followed by the same letters do not differ from each other at 1% (**) probability by the Scott Knott test.

**Table 2:** Germination speed index (GSI), germination percentage (%), normal, abnormal, and non-germinated seedlings of the three investigated *Brachiaria* cultivars after 13 days of germination.

Cultivars	GSI	Germination (%)	Abnormal	Non-Germination
Marandu	4.0 ± 0.57 a	70 ± 4.55 a	8 ± 1.31 a	22 ± 1.60 b
Piatã	4.3 ± 0.07 a	71 ± 1.73 a	12 ± 4.86 a	28 ± 1.31 b
Xaraés	2.3 ± 0.21 b	48 ± 4.99 b	14 ± 1.93 a	40 ± 1.89 a
**One-Way ANOVA**				
**F (*t*-test)**	30.3671 **	10.9039 *	**0.1614 ns**	9.1294 **
***p***	**<0.0001**	**0.0039**	**0.8534**	**0.0068**

Means ± SE (*n* = 4), Means followed by the same letters do not differ from each other at 1% (**) probability by the Scott Knott test; (ns) non-significant.

**Table 3:** X-ray image variables automatically analyzed using the ImageJ software.

Variables	Unit	Description
Area	mm^2^	Selection area obtained in square pixels and later converted to square millimeters.
Circularity	Circularity	Circularity = 4·π·Area/Perimeter^2^. Values of 1.0 indicate a perfect circle and values tending to 0 suggest an elongated shape.
Filling	%	Determined by dividing the area effectively filled with high-density tissue (gray levels above the initially defined threshold) by the total area of each seed.
Relative density	grey.pixel^−1^	Defined as the sum of the gray values of all pixels in a selected area divided by the number of pixels in the selection.
